# Self-rated health, subjective social status in school and socioeconomic status in adolescents: a cross-sectional study

**DOI:** 10.1186/s12889-019-7140-3

**Published:** 2019-06-20

**Authors:** Junia Joffer, Renée Flacking, Erik Bergström, Eva Randell, Lars Jerdén

**Affiliations:** 1 Department of Epidemiology and Global Health, Umeå University, SE-901 87 Umeå, Sweden; 2 Center for Clinical Research Dalarna-Uppsala University, Nissers väg 3, SE-791 82 Falun, Sweden; 3 School of Education, Health and Social Studies, Dalarna University, SE-791 88 Falun, Sweden; 4 Department of Clinical Sciences, Pediatrics, Umeå University, SE-901 87 Umeå, Sweden

**Keywords:** Adolescents, Gender, Health status, Self-rated health, Socioeconomic status, Subjective social status

## Abstract

**Background:**

Social position, traditionally measured by objective data on socioeconomic status (SES), is linked to health status in adults. In adolescents, the association is more uncertain and there are some studies suggesting that subjective social status (SSS) might be more adequate in relation to health. This study aimed to examine associations between SSS in school, SES and self-rated health (SRH) in adolescent boys and girls.

**Methods:**

A descriptive cross-sectional research design with quantitative survey data was used. The study involved 705 Swedish adolescents in upper secondary school (17–18-year-olds). SRH was measured with a single-item question and SSS by a question where adolescents were asked to assess their social position within their school. Formal education level of the parents was used as a proxy for objective SES. Univariable and multivariable ordinal regression analyses were conducted to assess the associations between SRH and SSS in school and SES.

**Results:**

In the multivariable analysis, SSS in school was positively associated with SRH, whereas no significant association between SES and SRH was found. The proportion of adolescents with high SRH increased with higher steps on the SSS ladder. Significant gender differences were found in that boys rated their SRH and SSS in school higher than girls did.

**Conclusions:**

The study shows that self-rated health in adolescents is related to perceived social position in school. Subjective social status in school seems to be a useful health-related measure of social position in adolescents.

## Background

Self-rated health (SRH), measured with a single-item question in which respondents are asked to assess their overall health [1, 2], is an established predictor of morbidity and mortality and hence an important health indicator. Negative trends of SRH in adolescents, and young people’s failure to achieve their full health potential [3] raise concerns and call for further research on the determinants of adolescent health. In this study health is defined as a subjective holistic construct in line with the WHO definition [4].

A socioeconomic gradient in health is a well-established and a widely accepted term in describing relationships between health outcome and socioeconomic status (SES). In adults and young children a positive association has consistently been demonstrated, i.e. higher SES is linked to less morbidity, lower risk of early mortality and better SRH [5, 6]. For adolescent health, however, the association is more uncertain, with some studies showing a positive association [7, 8] and others showing a weak or no association [9–12]. Traditionally, SES has been measured by objective measures, e.g. income, education or occupation. Over the past two decades, a growing body of research has challenged the use of these measures in favor of subjective indicators [13, 14]. Subjective social status (SSS) represents a person’s sense of position within a hierarchy. As an attempt to capture SSS, the MacArthur Network on SES and Health developed a scale consisting of a ladder with ten steps. In the early 2000s, youth versions of the scale were developed [15], one where adolescents are asked to assess their families’ placement in the society (i.e. societal SSS), and another where adolescents are asked to assess their own social position within their school community (i.e. SSS in school). The school ladder is based on such constructs as ‘respect’ and ‘standing’, which supposedly invites individuals to use the constructs as s/he sees fit when defining SSS [16]. SSS has been claimed to capture a more close youth perspective than socioeconomic measures, which primarily represent a parental perspective [15]. Thus, SSS may be a more comprehensive measure of social status than objective socioeconomic measures [13, 14, 17].

Few studies have investigated the association between SSS in school and SRH in adolescents; Page and colleagues [18] reported a positive association in adolescents from Central and Eastern Europe, as well as Diehl and colleagues [19] who found a positive association in German university students. Although studies on SSS in school and SRH are scarce, associations with other health-related variables and behaviors (e.g. depressive symptoms, weight status and smoking) have been identified [15, 16, 20]. Other studies investigating concepts similar to SSS in school have also shown an association to SRH. In a study among Swedish adolescents, Plenty and Mood demonstrated a relationship between low SRH and low ‘peer status’ [8]. A long-term effect of peer status has been shown in a Scottish cohort in which a positively graded association was found between higher peer status in adolescence and better SRH in mid-life [21]. When it comes to societal SSS, an association to SRH has been reported in adolescents in Finland [22], the U.S. [23], and Central and Eastern Europe [18].

Craig and colleagues conceive of adolescent SRH as a complex health indicator with multiple determinants [24]. In addition to subjective and objective social status indicators, variables such as gender, country of birth, family mood and parent relationships, self-esteem, body mass index (BMI), physical activity and smoking have also been linked to SRH [11, 18, 24–27].

The review of the current literature indicates that the association between social status and SRH in adolescents is not yet fully established and there is also a lack of consensus which measure of social status is the most appropriate to use among adolescents. In addition to this, the influence of traditionally used objective SES measures on adolescents’ health is inconclusive. Therefore, the aim of this study was to examine associations between SSS in school, SES and SRH in adolescent boys and girls.

## Methods

### Setting and sample

The study (Very Interesting Person, VIP) was conducted in three Swedish municipalities in central and northern Sweden, which represent varying numbers of inhabitants and levels of education in parents. Students from seven schools, covering the highest (*n* = 3) and lowest (*n* = 4) education levels of the parents in each municipality, were invited to answer a health questionnaire. All schools accepted the invitation. To date, four surveys have been performed in the VIP study, with the current study (conducted in 2008) using data from the last survey in upper secondary school (17–18-year-old adolescents). The size of the study sample was based on an estimation of statistical power concerning variables measuring empowerment [28]. In the fourth survey 705 students (318 boys and 387 girls), representing 67% of the eligible study population, answered a postal questionnaire that was sent home to the participants. The VIP study has been described in detail elsewhere [25, 28, 29].

### Questionnaire

A questionnaire was constructed to measure socio-demographic characteristics, inter- and intrapersonal aspects and health behavior. Most of the questionnaire items were derived from established Swedish surveys and the Swedish part of the HBSC study (including both mandatory and national questions) [30]. The questionnaire was tested for reliability (test-retest), which was performed during a pilot study. Items with kappa values < 0.40 were excluded [28]. In the fourth survey a question on SSS in school [15] was included.

#### Dependent variable

**Self-rated health** (SRH) was measured by the single-item question, “A person may feel good sometimes and bad sometimes. How do you feel most of the time?”. This question was rated on a scale with five response options (‘very good’, ‘rather good’, ‘neither good, nor bad’, ‘rather bad’ and ‘very bad’). Previous findings from a qualitative study with think-aloud interviews showed that adolescents recognize differences between response options [2]. Thus, all five response options were used when analyzing the data.

#### Covariates

**Subjective social status** (SSS) in school was examined using a youth version of the MacArthur Scale of Subjective Social Status, developed by Goodman and colleagues [15]. The question consists of a ladder with ten steps with the following text (slightly modified compared to Goodman and colleagues): “Assume that the ladder is a way of picturing your school. At the top of the ladder are the students with the most respect, who everyone wants to hang around with, and who have the highest standing. At the bottom of the ladder are the students who no one respects, no one wants to hang around with, and who have the lowest standing. Where would you place yourself on this ladder?” The variable sex/gender was measured by the question “Are you a boy or a girl?” In the study we recognize school as a social context in which gender structures are created [31]. In this context it is our belief that adolescents’ SRH is influenced by social constructions, rather than biological characteristics. Hence, we use the term **gender** when describing differences between boys and girls. **Country of birth** was measured by the question “In which country were you born?” The respondents were dichotomized into two groups (born in Sweden or abroad). **SES** was assessed by level of parental education, which was obtained from Statistics Sweden (the central government authority for official statistics), through their “Education registry”. This registry contains information about formal educational level of Swedish citizens. A data file was sent to Statistics Sweden where information about the parents’ educational level was added to the file. When dichotomized, families in which at least one of the parents had a college or university degree were defined as ‘high SES’, whereas ‘low SES’ included both compulsory school and upper secondary school. The variable **mood in family** [25] was assessed by the question “How do you consider the mood in your family?” The question was rated on a five-point ordinal scale and the answers of ‘very good’ and ‘rather good’ were merged as ‘good’. **Self-esteem** was measured by the question, “Do you like yourself?” A three-point ordinal scale was used as follows: ‘Yes, most often’, ‘Yes, sometimes’ and ‘No, seldom’, with ‘Yes, most often’ defined as ‘high self-esteem’, ‘Yes, sometimes’ and ‘No, seldom’ as ‘low self-esteem’. Self-reported weight and height was used to calculate **BMI** using the standard equation: BMI = weight (kg)/height (m)^2^. BMI was then categorized into four groups: underweight (< 18.5), normal weight (18.5–24.9), overweight (25–29.9) and obese (≥30). **Physical exercise** was assessed on a seven-point ordinal scale by the question, “How often do you usually exercise in your spare time (i.e. outside school) so you become breathless or sweating?” The answers of ‘two or three times weekly’ or ‘more often’ were merged as ‘high physical exercise’. **Smoking** was measured by the question, “How often do you smoke these days?” This question was rated on a four-point ordinal scale with the alternatives ‘every day’, ‘at least one time per week but not every day’, ‘less than one time per week’ and ‘I don’t smoke’. In the analyses a ‘smoker’ included all categories of smoking.

### Data analyses

First, descriptive statistics with chi-square tests was used to evaluate frequency differences between the groups. Then, in order to assess possible significant effects, gender specific multivariable ordinal regression with proportional odds was performed [32]. An ordinal regression accommodates an ordinal dependent variable (instead of a binary dependent variable used in binary logistic regression). In an ordinal regression analysis, the coefficients for the covariates (expressed as odds ratios) represent the odds of being one unit higher in the dependent variable associated with an increase of one unit in the covariate. In the multivariable analysis all available covariates theoretically potentially related to the dependent variable (SRH) were included, i.e. a full model. The ordinal regression model was carried out to test the importance of the pre-selected covariates, which is shown in the global significance test in the results. Finally, to further explore the relation between SSS in school and SRH, gender specific univariable ordinal regression analyses were applied in order to calculate model-based probabilities.

BMI was analyzed as a categorized variable in the descriptive analyses and as a continuous variable in the multivariable analyses. SSS in school was analyzed as an ordinal variable in the descriptive and univariable analyses, and as a continuous variable in the multivariable analyses. This choice was motivated to facilitate interpretations through the use of fewer parameters in the multivariable analysis, as the conclusions were the same regardless of how the two scales were analyzed. Missing data for the variables SRH, country of birth, SES and self-esteem was lower than 1%. For mood in the family, smoking and physical exercise missing data ranged from 1 to 2%. For BMI and SSS in school, missing data was 6.8 and 10.5% respectively. SPSS 24.0 was used as the analytical tool to conduct all descriptive statistics and the multivariable analyses. SAS 9.4 was used to calculate model-based probabilities. The significance level was set at *p* < 0.05 (two-sided).

## Results

Study variables stratified and compared by gender are shown in Table 1. The majority of the participants reported a rather good or very good SRH (boys 90.9%, girls 87.2%), with a significant gender difference (boys reported better SRH than girls, *p* < 0.01). Boys also rated their SSS in school (*p* = 0.02) and self-esteem (*p*<0.01) significantly higher than girls did, whereas girls reported more smoking (*p* = 0.02).

**Table 1:** Study characteristics by gender

	Boys (*N* = 318)	Girls (*N* = 387)	Chi-square *P*
	n (%)	n (%)	
Self-rated health			
Very bad	2 (0.6)	5 (1.3)	< 0.01
Rather bad	6 (1.9)	13 (3.4)	
Neither good, nor bad	21 (6.6)	31 (8.1)	
Rather good	146 (45.9)	215 (56.1)	
Very good	143 (45.0)	119 (31.1)	
Subjective social status in school			
1 (lowest)	2 (0.7)	2 (0.6)	0.02
2	–	3 (0.8)	
3	2 (0.7)	9 (2.5)	
4	11 (4.0)	13 (3.7)	
5	21 (7.6)	22 (6.2)	
6	52 (18.8)	68 (19.2)	
7	49 (17.7)	96 (27.1)	
8	75 (27.1)	87 (24.6)	
9	45 (16.2)	43 (12.1)	
10 (highest)	20 (7.2)	11 (3.1)	
Country of birth			
Born in Sweden	301 (94.7)	363 (94.3)	0.83
Born outside Sweden	17 (5.3)	22 (5.7)	
Socioeconomic status (parental education)			
High	177 (55.8)	202 (52.9)	0.44
Low	140 (44.2)	180 (47.1)	
Mood in family			
Good	262 (82.9)	301 (79.2)	0.22
Not good	54 (17.1)	79 (20.8)	
Self-esteem			
High	228 (72.2)	190 (49.6)	< 0.01
Low	88 (27.8)	193 (50.4)	
Body mass index			
Underweight	26 (8.4)	29 (8.3)	0.36
Normal weight	224 (72.7)	270 (77.4)	
Overweight	50 (16.2)	40 (11.5)	
Obesity	8 (2.6)	10 (2.9)	
Physical exercise			
High	187 (59.9)	239 (63.2)	0.38
Low	125 (40.1)	139 (36.8)	
Smoking			
Non-smokers	250 (79.4)	274 (71.5)	0.02
Smokers	65 (20.6)	109 (28.5)	

Gender specific multivariable ordinal regression analyses (Table 2) showed that an increase in SSS of one step on the ladder was associated with an increase in the odds of reporting a better SRH, regardless of gender: odds ratio [OR] = 1.53 (95% confidence interval [CI], 1.28–1.82) in boys and OR 1.45 (95% CI, 1.24–1.70) in girls. The analyses also showed that mood in family and self-esteem were positively and independently associated with SRH in both boys and girls. Furthermore, smoking was independently and negatively associated with SRH in boys. Being born in Sweden was positively associated with SRH in girls. We found no association between SES (parental education) and SRH.

**Table 2:** Ordinal regression analysis of self-rated health by gender

	Boys (*N* = 261)			Girls (*N* = 294)		
	OR	CI	*p*-value	OR	CI	*p*-value
Subjective social status in school^a^	1.53	1.28–1.82	< 0.01	1.45	1.24–1.70	< 0.01
Country of birth						
Born in Sweden	1.67	0.56–4.96	0.36	3.85	1.44–10.28	0.01
Born outside Sweden	1.00	Ref		1.00	Ref	
Socioeconomic status (parental education)						
High	0.90	0.53–1.51	0.68	1.42	0.88–2.28	0.15
Low	1.00	Ref		1.00	Ref	
Mood in family						
Good	3.53	1.62–7.67	< 0.01	1.96	1.10–3.51	0.02
Not good	1.00	Ref		1.00	Ref	
Self-esteem						
High	3.15	1.61–6.15	< 0.01	3.73	2.17–6.40	< 0.01
Low	1.00	Ref		1.00	Ref	
Body mass index^a^	1.00	0.92–1.08	0.96	0.94	0.87–1.02	0.14
Physical exercise						
High	1.26	0.73–2.16	0.41	1.35	0.82–2.22	0.24
Low	1.00	Ref		1.00	Ref	
Smoking						
Non-smokers	2.30	1.16–4.55	0.02	0.95	0.54–1.68	0.86
Smokers	1.00	Ref		1.00	Ref	

^a^Continuous variable

**Fig. 1:**
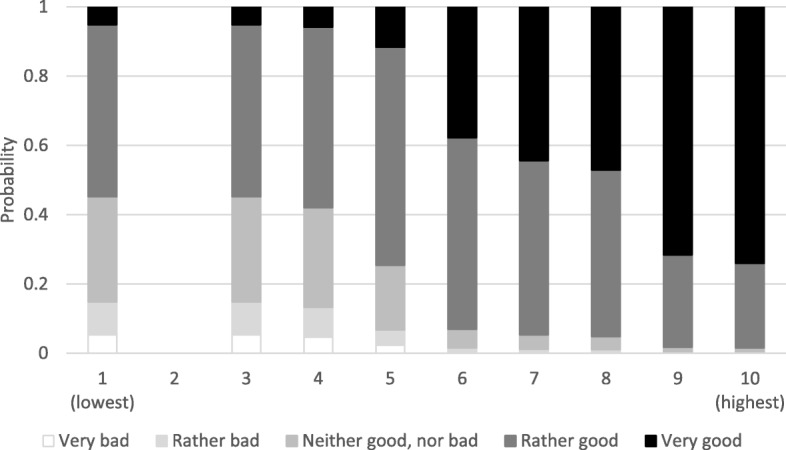
Univariable predicted cumulative probabilities of SSS in school and SRH in boys (*n* = 277)

**Fig. 2:**
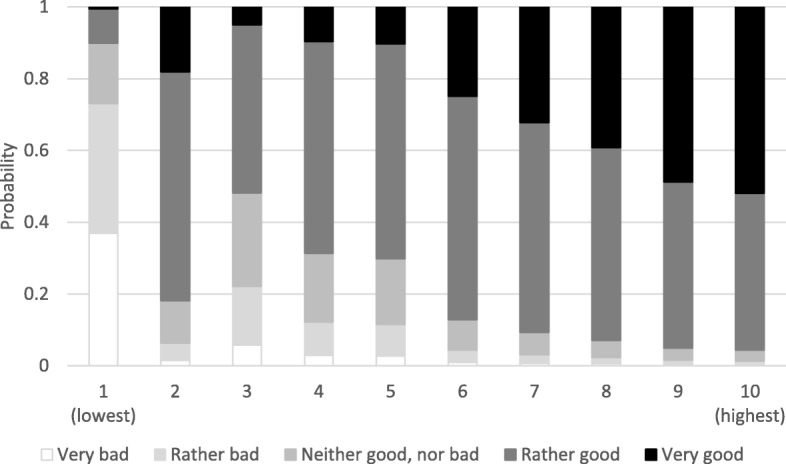
Univariable predicted cumulative probabilities of SSS in school and SRH in girls (*n* = 351)

When exploring the association between SSS in school and SRH further, univariable predicted cumulative probabilities showed a linear association, implying that higher steps on the SSS scale increased the likelihood of reporting better SRH in boys (Fig. 1) and girls (Fig. 2).

## Discussion

Our main findings were that 1) SSS in school was positively related to adolescents’ SRH in an analysis controlling for pre-selected covariates and 2) at this age, SES (parental education) was not associated with SRH in Swedish adolescents.

### SSS in school and SRH

The positive association between SSS in school and SRH found among both boys and girls, could be explained from the perspective that adolescence represents a period in life when peer group approval and search for belonging play an integral part of the everyday life of adolescents [21, 33, 34]. While previous studies [2, 34] have reported that relational and social aspects are prominent in adolescents’ conceptualization of SRH, the association between SSS in school (being a social construct) and SRH might not be surprising.

Studying the relation between SSS in school and SRH, Page and colleagues [18] found an association in Central and Eastern European adolescents. The study included two SSS scales, namely the societal SSS and SSS in school. Page and colleagues observed a univariable significant association between SSS in school and SRH. In a multivariable analysis, however, a significant association was only detected for societal SSS. It is plausible that the strong association between societal SSS and SRH represents a more pronounced family-oriented tradition in Central and Eastern Europe. If so, indicators based on the social position of the family might be more important to adolescents’ health in Central and Eastern Europe than indicators solely tied to an adolescent context.

Diehl and colleagues [19] noted a similar association in German university students who were asked to assess two SSS scales, i.e. their perceived social standing with reference to other university students in Germany, and societal SSS (i.e. the general population in Germany). Diehl and colleagues reported associations for both scales though the strongest association for societal SSS. This might be explained by the older age of their study population (mean age 22.69 years). For individuals entering young adulthood, measures linked to objective SES indicators are likely to become more influential.

Although studies on SSS in school and SRH are sparse, a resembling construct, namely ‘peer status’, has also been linked to SRH [8, 21]. Peer status, as measured through sociometric nominations (e.g., adolescents are asked to list the pupils in the class they like the best, or the most popular persons in the class), show that schools are an important setting for creating and confirming social positions. Adolescents compare themselves with other adolescents and perform subjective assessments. However, because the listing of peers may differ from a subjective assessment of one’s own positioning among peers, comparisons should be made with caution.

Our finding of a significant gender difference for SRH (boys rating their SRH higher than girls) is in line with previous studies [18, 27]. For SSS in school, boys also rated a higher status than girls, which differs from adolescents in the USA, as reported by Goodman and colleagues [15]. It should be noted that a slightly different wording of the SSS question was used in this study. Goodman and colleagues embedded the term ‘grades’ in their question, this was not included in the Swedish version. In general, girls achieve better academic grades than boys, which might explain girls’ perception of higher placement in the sample of adolescents in the USA. It is possible that the gender difference in SRH and SSS found in our study could partly be influenced by the fact that adolescent boys, as a demonstration of toughness and masculinity, are more likely to respond with higher ratings than adolescent girls [35]. Another contributing explanation might be related to power relations in society that reflect a male advantage [36].

### SES and SRH

We found no association between SRH and parental SES, supporting the theory of a socioeconomic equalization in health outcome [37]. A feasible explanation for this theory is that adolescence is a transition period during which, among other things, parental influence decreases and autonomy increases [38].

It should be noted, that while our study used parental education as a proxy for SES, others have argued that economic and material resources may provide social status to adolescents in a more evident way [7, 8]. Plenty and Mood demonstrated associations between parental occupation and income and SRH, but not between parental education level and SRH. Although educational attainment is considered a valid measure in adults [39], it would have been beneficial if information about additional SES measures had been available in our study. However, regardless of the relation between SES and adolescents’ SRH, social position among peers seems to be important in relation to adolescents’ health.

The lack of a significant association between SES and SRH is supported by Siahpush and Sing [11], who reported similar results for parental education and occupation in adolescents in Australia. Furthermore, Sweeting and Hunt [40] noted that family affluence, residential depravation and societal SSS had weaker associations with health and wellbeing compared with different dimensions of school-based SSS (i.e. ‘SSS-peer’, ‘SSS-scholastic’ and ‘SSS-sports’). However, because a different proxy for health was used (physical symptoms, psychological distress and anger), the results are not fully comparable with our study. Nevertheless, Sweeting and Hunt emphasize the importance of studying school-based SSS in adolescence [40].

### Methodological considerations

One strength of our study is that data were collected using a postal questionnaire. Postal questionnaires are likely to offer a high level of confidentiality and by that more honest responses [2]. We regard the response rate of 67% of all eligible students at the participating schools as acceptable. Data on parental education were obtained from Statistics Sweden, a central government authority for official statistics and other government statistics, a highly reliable data source [41]. In the analysis, parental educational level was dichotomized. A trichotomization was considered, yet not chosen since the group “compulsory school” was rather small (boys *n* = 13 and girls *n* = 12, in the multivariable model). The use of an ordinal regression analysis enabled us to analyze all five response options of the question pertaining to SRH. Previous research [2] has shown that adolescents differentiate between all the response options of the SRH question. Finally, the multivariable analysis included important variables potentially influencing SRH, covering socio-demographics, inter- and intrapersonal variables and behavioral factors. Although SRH certainly is influenced by factors in addition to those evaluated in the study, we regard the multivariable analysis as a strength.

The study has some limitations. First, the cross-sectional research design establishes associations but not causations, limiting the conclusions that can be drawn from the study. Second, there is the risk of selection bias in the sample. More specifically, adolescents with the lowest ratings of SSS in school or SRH might not answer the questionnaire. It should also be noted that potentially important factors such as societal SSS and parental income were not included in the study. While mainly using subjective measures, the respondents’ personalities should be acknowledged as a possible confounder. Finally, because the study was conducted in a high-income country, this may potentially limit the generalizability. The study was performed in three municipalities, which, according to official statistics, are fairly representative of Sweden with respect to SES. Therefore, we believe that the results can be generalized to Swedish adolescents in general.

### Implications

The results indicate that SSS in school, i.e. the subjective social position among peers, may be a useful health related measure of social position in adolescence. Further studies are needed to continue investigating the association between SSS in school, SRH and various SES measurements in other youth populations. Longitudinal studies are called for to address the question of causality. Furthermore, qualitative studies may enable the exploration of adolescents’ perception of different SSS constructs.

## Conclusions

The study shows that self-rated health in adolescents is related to perceived social position in school. Subjective social status in school seems to be a useful health-related measure of social position in adolescents.

## Data Availability

Data is available for other researchers in an unidentified form, after agreement with the research group.

## Abbreviations

SES: Socioeconomic status
SRH: Self-rated health
SSS: Subjective social status

## References

[1] Fayers PM, Sprangers MA (2002). Understanding self-rated health. Lancet.

[2] Joffer J, Jerdén L, Öhman A, Flacking R (2016). Exploring self-rated health among adolescents: a think-aloud study. BMC Public Health.

[3] Currie C, Zanotti C, Morgan A, Currie D, de Looze M, Roberts C, Samdal O, Smith ORF, Barnekow V (2012). Social determinants of health and well-being among young people.

[4] World Health Organization (1948). Constitution of the World Health Organization.

[5] Marmot M (2005). Social determinants of health inequalities. Lancet.

[6] Kennedy BP, Kawachi I, Glass R, Prothrow-Stith D (1998). Income distribution, socioeconomic status, and self rated health in the United States: multilevel analysis. BMJ.

[7] Richter M, Moor I, van Lenthe FJ (2012). Explaining socioeconomic differences in adolescent self-rated health: the contribution of material, psychosocial and behavioural factors. J Epidemiol Community Health.

[8] Plenty S, Mood C (2016). Money, peers and parents: social and economic aspects of inequality in youth wellbeing. J Youth Adolesc.

[9] Piko B, Fitzpatrick KM (2001). Does class matter? SES and psychosocial health among Hungarian adolescents. Soc Sci Med.

[10] Goodman E, Amick BC, Rezendes MO, Tarlov AR, Rogers WH, Kagan J (1997). Influences of gender and social class on adolescents’ perceptions of health. Arch Pediatr Adolesc Med.

[11] Siahpush M, Singh G (2000). A multivariate analysis of the association between social class of origin and current social class with self-rated general health and psychological health among 16-year-old Australians. Aust NZ J Med.

[12] Spencer NJ (2006). Social equalization in youth: evidence from a cross-sectional British survey. Eur J Pub Health.

[13] Quon EC, McGrath JJ (2013). Subjective socioeconomic status and adolescent health: a meta-analysis. Health Psychol.

[14] Präg P, Mills MC, Wittek R (2016). Subjective socioeconomic status and health in cross-national comparison. Soc Sci Med.

[15] Goodman E, Adler NE, Kawachi I, Frazier AL, Huang B, Colditz GA (2001). Adolescents’ perceptions of social status: development and evaluation of a new indicator. Pediatrics.

[16] Goodman E, Adler NE, Daniels SR, Morrison JA, Slap GB, Dolan LM (2003). Impact of objective and subjective social status on obesity in a biracial cohort of adolescents. Obesity Res.

[17] Euteneuer F (2014). Subjective social status and health. Curr Opin Psychiatry.

[18] Page RM, Simonek J, Ihász F, Hantiu I, Uvacsek M, Kalabiska I, Klarova R (2009). Self-rated health, psychosocial functioning, and other dimensions of adolescent health in Central and Eastern Europe adolescents. Eur J Psychiat.

[19] Diehl K, Hoebel J, Sonntag D, Hilger J. Subjective social status and its relationship to health and health behavior: comparing two different scales in university students. Int J Adolesc Med Health. 2017. 10.1515/ijamh-2017-0079. 10.1515/ijamh-2017-0079 28841574

[20] Finkelstein DM, Kubzansky LD, Goodman E (2006). Social status, stress, and adolescent smoking. J Adolesc Health.

[21] Östberg V, Modin B (2008). Status relations in school and their relevance for health in a life course perspective: findings from the Aberdeen children of the 1950’s cohort study. Soc Sci Med.

[22] Karvonen S, Rahkonen O (2011). Subjective social status and health in young people. Sociol Health Illn.

[23] Goodman E, Huang B, Schafer-Kalkhoff T, Adler NE (2007). Perceived socioeconomic status: a new type of identity that influences adolescents’ self-rated health. J Adolesc Health.

[24] Craig BA, Morton DP, Morey PJ, Kent LM, Gane AB, Butler TL, Rankin PM, Price KR (2018). The association between self-rated health and social environments, health behaviors and health outcomes: a structural equation analysis. BMC Public Health.

[25] Jerdén L, Burell G, Stenlund H, Weinehall L, Bergström E (2011). Gender differences and predictors of self-rated health development among Swedish adolescents. J Adolesc Health.

[26] Lindström M, Moden B, Rosvall M (2014). Country of birth, parental background and self-rated health among adolescents: a population-based study. Scand J Public Health.

[27] Wade TJ, Pevalin DJ, Vingilis E (2000). Revisiting student self-rated physical health. J Adolesc.

[28] Jerdén L (2007). Health promoting health services: personal health documents and empowerment. Doctoral thesis.

[29] Joffer J, Burell G, Bergström E, Stenlund H, Sjörs L, Jerdén L (2014). Predictors of smoking among Swedish adolescents. BMC Public Health.

[30] Danielson M, Marklund U (2000). Svenska skolbarns hälsovanor 1997/98 (Health behaviours in school-aged children 1997/98).

[31] Connell R (1996). Teaching the boys: new research on masculinity, and gender strategies for schools. Teach Coll Rec.

[32] Agresti A (2003). Categorical data analysis.

[33] Flacking R, Jerdén L, Bergström E, Starrin B (2014). ‘In or out’—on the dynamic between acceptance and rejection and its influence on health in adolescent girls. Young.

[34] Randell E, Jerdén L, Öhman A, Flacking R (2016). What is health and what is important for its achievement? A qualitative study on adolescent boys’ perceptions and experiences of health. Open Nurs J.

[35] Randell E, Jerdén L, Öhman A, Starrin B, Flacking R (2016). Tough, sensitive and sincere: how adolescent boys manage masculinities and emotions. Int J Adolesc Youth.

[36] Connell RW, Messerschmidt JW (2005). Hegemonic masculinity: rethinking the concept. Gend Soc.

[37] West P, Sweeting H (2004). Evidence on equalisation in health in youth from the West of Scotland. Soc Sci Med.

[38] Sebald H (1992). Adolescence : a social psychological analysis.

[39] Padilla-Moledo C, Ruiz J, Castro-Piñero J (2016). Parental educational level and psychological positive health and health complaints in Spanish children and adolescents. Child Care Health Dev.

[40] Sweeting H, Hunt K (2014). Adolescent socio-economic and school-based social status, health and well-being. Soc Sci Med.

[41] Statistics Sweden (2018). Statistics Sweden.

